# Impact of Early COVID‐19 Antiviral Therapy on the Incidence of Uveitis: A Retrospective Cohort Study Using the TriNetX Database

**DOI:** 10.1002/iid3.70455

**Published:** 2026-05-06

**Authors:** Hou‐Ting Kuo, Alan Y. Hsu, De‐Yi Liu, Bing‐Qi Wu, James Cheng‐Chung Wei, Ning‐Yi Hsia, Yi‐Ching Shao, Chun‐Ting Lai, Chun‐Chi Chiang, Chun‐Ju Lin, Huan‐Sheng Chen, Yu‐Hsun Wang, Hsin Tseng, Ho‐Che Hsu, Yi‐Yu Tsai

**Affiliations:** ^1^ Department of Ophthalmology, China Medical University Hospital China Medical University Taichung Taiwan; ^2^ Department of General Medicine China Medical University Hospital Taichung Taiwan; ^3^ Department of Allergy, Immunology & Rheumatology Chung Shan Medical University Hospital Taichung Taiwan; ^4^ Graduate Institute of Integrated Medicine China Medical University Taichung Taiwan; ^5^ Department of Nursing, Institute of Medicine Chung Shan Medical University Taichung Taiwan; ^6^ School of Medicine, College of Medicine China Medical University Taichung Taiwan; ^7^ Department of Optometry Asia University Taichung Taiwan; ^8^ Graduate Institute of Biomedical Sciences, College of Medicine China Medical University Taichung Taiwan; ^9^ An‐Shin Dialysis Center, Excelsior Renal Service Co. Ltd. Taiwan Branch Taichung Taiwan; ^10^ Department of Medical Research Chung Shan Medical University Hospital Taichung Taiwan

**Keywords:** antiviral, COVID‐19, uveitis

## Abstract

**Objective:**

To assess whether antivirals are associated with a reduced incidence of uveitis following COVID‐19.

**Methods:**

We conducted a multi‐institutional, population‐based retrospective cohort study of adults (≥ 18 years) diagnosed with COVID‐19 between 2022 and 2024. Patients who received antiviral agents (Paxlovid, Molnupiravir, or Remdesivir) within 5 days of diagnosis were matched 1:1 with untreated controls using propensity score matching. Patients with pre‐existing uveitis, early‐onset uveitis within 5 days of the index date, or underlying systemic inflammatory or infectious diseases were excluded. The primary outcome was new‐onset uveitis, with hazard ratios (HRs) calculated across follow‐up intervals.

**Results:**

After matching, 438,455 patients were included in both the antiviral and non‐antiviral groups. Antiviral therapy was associated with a significantly lower risk of uveitis at 3 months (HR = 0.62, 95% CI: 0.45–0.87), 6 months (HR = 0.68, 95% CI: 0.54–0.87), 1 year (HR = 0.76, 95% CI: 0.64–0.91), 3 years (HR = 0.80, 95% CI: 0.70–0.92), and all duration (HR = 0.81, 95% CI: 0.71–0.93). Subgroup analysis revealed consistent benefit across all age groups, with females experiencing greater protection than males. Significant reductions in uveitis risk were observed among patients with diabetes (HR = 0.68, 95% CI: 0.52–0.89), hyperlipidemia (HR = 0.78, 95% CI: 0.65–0.95), and heart failure (HR = 0.52, 95% CI: 0.30–0.90). Among the antivirals, Paxlovid was associated with a significant risk reduction (HR = 0.83, 95% CI: 0.71–0.96), whereas Molnupiravir and Remdesivir showed no statistically significant effect. CEV classification did not show significant improvement. Besides, the risk reduction was evident regardless of prior COVID‐19 vaccination status.

**Conclusions:**

Early antiviral treatment for COVID‐19 such as Paxlovid, is associated with a reduced risk of uveitis. These findings suggest that, in addition to mitigating systemic disease progression, antiviral therapy may confer ocular protective effects, which could be especially meaningful for high‐risk populations.

## 1 Introduction

Uveitis encompasses a diverse group of intraocular inflammatory disorders primarily affecting the iris, ciliary body, and choroid. As a leading cause of visual impairment in working‐age populations, it accounts for approximately 10%–15% of blindness cases in developed nations [1]. Its etiology is multifactorial, spanning autoimmune, infectious, traumatic, and idiopathic origins. Despite therapeutic advancements, the recurrent nature of uveitis and the risk of irreversible vision loss remain persistent clinical and public health challenges [2, 3].

Since the onset of the COVID‐19 pandemic, the extrapulmonary manifestations of SARS‐CoV‐2 infection have garnered increasing attention [1, 2]. Among these, the ocular system has emerged as a potential target for both direct viral invasion and immune‐mediated inflammatory responses [6]. While conjunctivitis is the most frequently reported ocular manifestation of COVID‐19, accumulating evidence has suggested a possible association between SARS‐CoV‐2 infection and the development of uveitis, observed both during the acute phase of infection and in the post‐infectious period [6, 7]. Proposed mechanisms include direct viral entry via ocular ACE2 receptors, deposition of immune complexes, and cytokine‐driven inflammatory responses resembling those observed in multisystem inflammatory syndrome [4, 6, 7].

In response to the global health crisis, several antiviral agents have been developed and authorized for clinical use to mitigate the severity and progression of COVID‐19 [8]. Among the most widely used agents are nirmatrelvir/ritonavir (Paxlovid), molnupiravir, and remdesivir, each exhibiting unique mechanisms of action. Paxlovid inhibits the SARS‐CoV‐2 main protease (Mpro), thereby blocking viral replication; molnupiravir serves as a nucleoside analog that interferes with viral RNA synthesis through lethal mutagenesis; and remdesivir acts as a viral RNA‐dependent RNA polymerase inhibitor [9, 10, 11]. When administered early during infection, these antiviral agents have been shown to significantly reduce hospitalization and mortality, particularly in high‐risk populations with underlying comorbidities or immunosuppression [12, 13]. However, large‐scale studies examining the association between antiviral therapy for COVID‐19 and the subsequent risk of developing uveitis are currently lacking.

Given the growing recognition of long‐term sequelae following COVID‐19 and the potential immunopathogenic link between systemic viral inflammation and uveitis, we conducted a large‐scale retrospective cohort study using the TriNetX global health research network. We examined whether early administration of Paxlovid, molnupiravir, or remdesivir in adults with confirmed COVID‐19 was associated with a reduced incidence of subsequent uveitis, with prespecified subgroup analyses according to demographic and clinical characteristics. We hypothesized that early antiviral treatment for COVID‐19 is associated with a lower risk of new‐onset uveitis.

## 2 Materials and Methods

### 2.1 Data Source and Study Design

This retrospective cohort study was conducted using the TriNetX Analytics Platform, a federated health research network that provides access to de‐identified electronic medical records from participating healthcare organizations, primarily in the United States. The platform contains real‐world data on diagnoses, procedures, medications, and laboratory results. The use of this database is compliant with the Health Insurance Portability and Accountability Act. Given the de‐identified nature of the data, the study received an exemption from a full review by the Institutional Review Board of Chung Shan Medical University Hospital. This study was conducted and reported in accordance with the Strengthening the Reporting of Observational Studies in Epidemiology (STROBE) guidelines. This study was approved by the Institutional Review Board of the Chung Shan Medical University Hospital Research Ethics Committee (CMUHCS2‐21176).

### 2.2 Study Population and Cohort Definition

**Figure 1:**
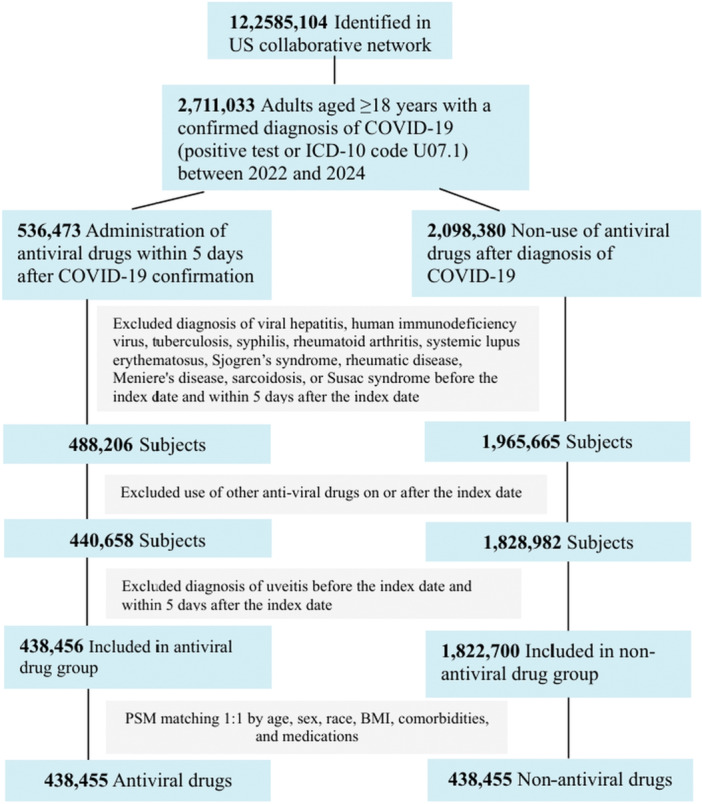
Subject selection flowchart.

The patient selection process is detailed in the flowchart (Figure 1). We identified 2,711,033 adult patients (≥ 18 years) with a confirmed diagnosis of COVID‐19 between January 1, 2022, and December 31, 2024, within the TriNetX U.S. network. A confirmed diagnosis was defined by either a positive laboratory test for SARS‐CoV‐2 or the presence of the International Classification of Diseases, Tenth Revision, Clinical Modification (ICD‐10‐CM) code U07.1.

The exposure cohort included patients who received antiviral therapy (ritonavir‐nirmatrelvir [Paxlovid], molnupiravir, or remdesivir) within 5 days of their COVID‐19 diagnosis. The date of the COVID‐19 diagnosis was set as the index date. The comparator cohort consisted of patients with COVID‐19 who did not receive any of the specified antiviral treatments after the index date.

To ensure the analysis focused on new‐onset uveitis, patients with a pre‐existing diagnosis of uveitis or a new diagnosis within 5 days after the index date were excluded. Additional exclusion criteria for both cohorts included prior diagnoses of viral hepatitis, human immunodeficiency virus, tuberculosis, syphilis, rheumatoid arthritis, systemic lupus erythematosus, Sjögren's syndrome, rheumatic disease, Menière's disease, sarcoidosis, or Susac syndrome. Patients who used other systemic antiviral agents after the index date were also excluded. The specific codes used for cohort definition are listed in Tables S1–S3.

### 2.3 Covariates and Propensity Score Matching (PSM)

We collected baseline data on demographics (age, sex, race, ethnicity, body mass index [BMI]) and clinical variables, including comorbidities and medications prescribed within the 12 months prior to the index date. To minimize confounding, we performed 1:1 PSM between the antiviral and non‐antiviral cohorts. The propensity scores were calculated using a logistic regression model based on age, sex, race, BMI, and the presence of pre‐existing comorbidities and medication use as listed in Table 1. A greedy nearest‐neighbor matching algorithm with a caliper of 0.25 was utilized to balance the cohorts. We assessed covariate balance using standardized mean differences (SMDs), with a value < 0.1 considered indicative of a negligible difference between the groups.

**Table 1:** Demographic characteristics of antiviral and non‐antiviral.

	Before PSM			After PSM		
	Antiviral, *N* = 438,456	Non‐antiviral, *N* = 1,822,700	SMD	Antiviral, *N* = 438,455	Non‐antiviral, *N* = 438,455	SMD
Age, Mean ± SD	60.21 ± 16.48	49.77 ± 18.88	0.589	60.21 ± 16.48	60.44 ± 16.81	0.014
Sex						
Female	243,305 (55.49)	1,070,187 (58.71)	0.065	243,304 (55.49)	243,989 (55.65)	0.003
Male	180,833 (41.24)	705,822 (38.72)	0.051	180,833 (41.24)	179,831 (41.02)	0.005
Unknown gender	14,318 (3.27)	46,691 (2.56)	0.042	14,318 (3.27)	14,635 (3.34)	0.004
Ethnicity						
Not Hispanic or Latino	318,933 (72.74)	1,217,780 (66.81)	0.129	318,932 (72.74)	318,061 (72.54)	0.004
Hispanic or Latino	26,515 (6.05)	153,138 (8.40)	0.091	26,515 (6.05)	25,756 (5.87)	0.007
Unknown Ethnicity	93,008 (21.21)	451,782 (24.79)	0.085	93,008 (21.21)	94,638 (21.58)	0.009
Race						
White	320,890 (73.19)	1,169,670 (64.17)	0.195	320,890 (73.19)	324,570 (74.03)	0.019
Black or African American	43,373 (9.89)	292,725 (16.06)	0.184	43,373 (9.89)	43,529 (9.93)	0.001
Asian	24,529 (5.59)	86,719 (4.76)	0.038	24,528 (5.59)	22,885 (5.22)	0.017
Native Hawaiian or other Pacific Islander	3130 (0.71)	17,075 (0.94)	0.025	3130 (0.71)	3223 (0.74)	0.003
American Indian or Alaska Native	1681 (0.38)	8000 (0.44)	0.009	1681 (0.38)	1652 (0.38)	0.001
Other race	11,898 (2.71)	80,588 (4.42)	0.092	11,898 (2.71)	10,884 (2.48)	0.015
Unknown race	32,955 (7.52)	167,923 (9.21)	0.061	32,955 (7.52)	31,712 (7.23)	0.011
BMI, Mean ± SD	30.35 ± 7.50	30.25 ± 7.65	0.014	30.35 ± 7.50	30.01 ± 7.25	0.046
Comorbidities						
Hypertension	177,493 (40.48)	440,857 (24.19)	0.354	177,492 (40.48)	177,149 (40.40)	0.002
Diabetes mellitus	77,337 (17.64)	202,399 (11.10)	0.187	77,336 (17.64)	73,822 (16.84)	0.021
Ischemic heart diseases	48,529 (11.07)	123,264 (6.76)	0.152	48,529 (11.07)	47,041 (10.73)	0.011
Hyperlipidemia	180,668 (41.21)	399,143 (21.90)	0.425	180,667 (41.21)	177,230 (40.42)	0.016
Heart failure	23,195 (5.29)	69,290 (3.80)	0.072	23,195 (5.29)	22,597 (5.15)	0.006
Cerebral infarction	7357 (1.68)	23504 (1.29)	0.032	7357 (1.68)	6889 (1.57)	0.008
Medications						
Ophthalmologicals	197,668 (45.08)	611,701 (33.56)	0.238	197,667 (45.08)	197,257 (44.99)	0.002
Corticosteroids for systemic use	140,616 (32.07)	409,410 (22.46)	0.217	140,615 (32.07)	139,800 (31.89)	0.004
Acetaminophen	89,773 (20.48)	319,480 (17.53)	0.075	89,773 (20.48)	89,692 (20.46)	0.000
Anti‐inflammatory and antirheumatic products, non‐steroids	89,896 (20.50)	314,014 (17.23)	0.084	89,895 (20.50)	88,099 (20.09)	0.010
Aspirin	37,581 (8.57)	117,245 (6.43)	0.081	37,581 (8.57)	36,611 (8.35)	0.008
Metformin	37,756 (8.61)	80,972 (4.44)	0.169	37,755 (8.61)	34,699 (7.91)	0.025
Bevacizumab	714 (0.16)	1803 (0.10)	0.018	714 (0.16)	612 (0.14)	0.006
Interferon beta‐1a	75 (0.02)	141 (0.01)	0.008	75 (0.02)	40 (0.01)	0.007
Fingolimod	59 (0.01)	178 (0.01)	0.003	59 (0.01)	27 (0.01)	0.007
Tocilizumab	37 (0.01)	627 (0.03)	0.018	37 (0.01)	111 (0.03)	0.013
Sarilumab	15 (0.00)	40 (0.00)	0.002	15 (0.00)	19 (0.00)	0.001

Abbreviation: SMD, standardized mean difference.

### 2.4 Outcome Measures

The primary outcome was the incidence of new‐onset uveitis after the 5‐day exclusionary period following the index date, identified using ICD‐10‐CM codes. We also analyzed the risk of specific uveitis subtypes, including iridocyclitis, focal chorioretinal inflammation, posterior cyclitis, retinal vasculitis, and panuveitis. Outcomes were assessed at multiple follow‐up intervals: 3 months, 6 months, 1 year, 2 years, and 3 years, as well as for the entire follow‐up period. Subgroup analyses were performed based on age, sex, race, BMI, specific comorbidities, COVID‐19 vaccination status, type of antiviral drug, and classification as clinically extremely vulnerable (CEV).

### 2.5 Statistical Analysis

**Figure 2:**
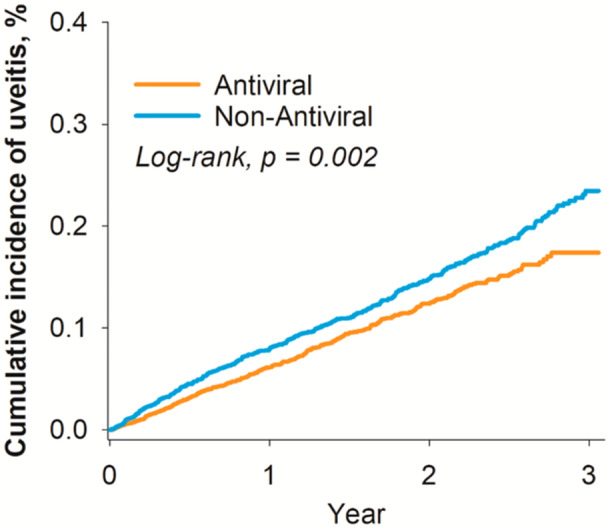
Kaplan–Meier analysis for risk of uveitis.

Baseline characteristics of the two cohorts were compared using SMDs before and after PSM. We used the Kaplan–Meier method to estimate the cumulative incidence of uveitis, and the log‐rank test to compare the survival curves (Figure 2). Crude Cox proportional hazards models based solely on the matched design were used to estimate hazard ratios (HRs) and corresponding 95% confidence intervals (CIs) for the association between antiviral use and the risk of uveitis. A two‐sided *p*‐value < 0.05 was considered statistically significant. All statistical analyses were conducted using the built‐in functionalities of the TriNetX platform.

## 3 Results

### 3.1 Baseline Characteristics of the Study Cohort

After 1:1 PSM, the final study population consisted of 438,455 patients in the antiviral group and 438,455 patients in the non‐antiviral group. Baseline characteristics were well‐balanced after matching, with all SMDs falling below the 0.1 threshold (Table 1). The mean age of the matched cohorts was 60.21 ± 16.48 years in the antiviral group and 60.44 ± 16.81 years in the non‐antiviral group. In both groups, approximately 55.5% of patients were female. The racial distribution was predominantly White (73.2% in the antiviral group vs. 74.0% in the non‐antiviral group), followed by Black or African American (9.9% vs. 9.9%), and Asian (5.6% vs. 5.2%). Common comorbidities, such as hypertension (40.5% vs. 40.4%), diabetes mellitus (17.6% vs. 16.8%), and hyperlipidemia (41.2% vs. 40.4%), were equally distributed. Prior medication use was also comparable between the groups.

### 3.2 Risk of Uveitis Associated With Antiviral Use

Over the entire follow‐up period, the cumulative incidence of uveitis was significantly lower in the antiviral group (391 events, 0.089%) compared to the non‐antiviral group (479 events, 0.110%), with an overall HR of 0.81 (95% CI: 0.71–0.93) (Table 2). This risk reduction was consistent and statistically significant across all measured time points, with the most pronounced effect observed within the first 3 months of follow‐up (HR = 0.62; 95% CI: 0.45–0.87). The Kaplan–Meier curves in Figure 2 visually demonstrate the lower cumulative risk of uveitis in the antiviral cohort.

**Table 2:** Risk of uveitis exposed to antiviral compared to non‐antiviral.

	No. of uveitis		
	Antiviral, *N* = 438,455	Non‐antiviral, *N* = 438,455	HR (95% CI)
Follow‐up duration			
3 months	56	87	**0.62 (0.45–0.87)**
6 months	115	162	**0.68 (0.54–0.87)**
1 year	211	264	**0.76 (0.64–0.91)**
2 years	345	404	**0.82 (0.71–0.94)**
3 years	388	476	**0.80 (0.70–0.92)**
All	391	479	**0.81 (0.71–0.93)**
Iridocyclitis	370	450	**0.82 (0.71–0.94)**
Focal chorioretinal inflammation	10	10	0.25 (0.05–1.17)
Unspecified disseminated chorioretinal inflammation	0	10	N/A
Posterior cyclitis	12	13	0.94 (0.43–2.07)
Retinal vasculitis	14	14	0.95 (0.45–2.00)
Panuveitis	10	10	0.69 (0.26–1.81)

*Note:* If the patient's count is 1–10, the results indicate a count of 10. Bold values are statistical significance.

Abbreviation: N/A, not applicable.

Among uveitis subtypes, the use of antivirals was associated with a significantly lower risk of iridocyclitis (HR = 0.82; 95% CI: 0.71–0.94). No statistically significant associations were found for other subtypes, such as posterior cyclitis, retinal vasculitis, or panuveitis.

### 3.3 Subgroup Analyses

**Figure 3:**
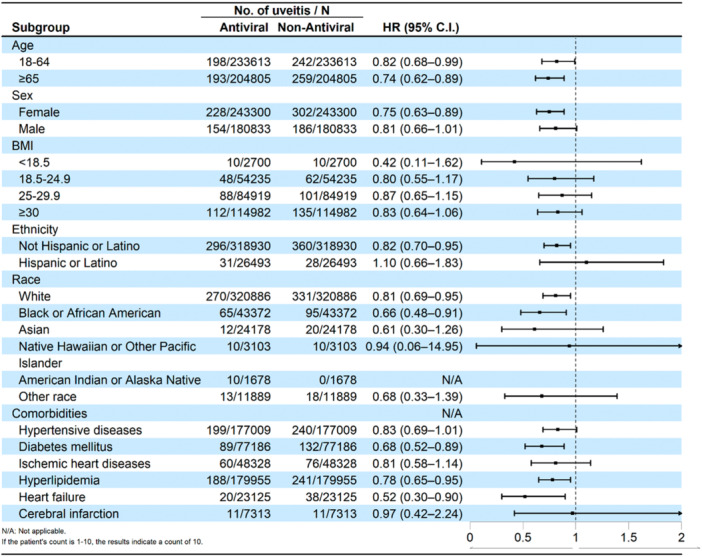
Forest plot of the risk of uveitis exposed to antiviral compared to non‐antiviral.

Forest plot of the risk of uveitis exposed to antiviral compared to non‐antiviral was showed at Figure 3. Subgroup analyses revealed that the protective association of antiviral use was particularly evident in certain populations (Tables 3 and 4). A significantly lower risk was observed in patients aged ≥ 65 years (HR = 0.74; 95% CI: 0.62–0.89) and those aged 18‐64 (HR = 0.82; 95% CI: 0.68–0.99). Among females, the risk was also significantly reduced (HR = 0.75; 95% CI: 0.63–0.89), whereas the association in males did not reach statistical significance (HR = 0.81; 95% CI: 0.66–1.01).

**Table 3:** Subgroup analysis of risk of uveitis exposed to antiviral compared to non‐antiviral.

	Antiviral		Non‐antiviral		
	*N*	No. of event	*N*	No. of event	HR (95% CI)
Age					
18–64	233,613	198	233,613	242	**0.82 (0.68–0.99)**
≥ 65	204,805	193	204,805	259	**0.74 (0.62–0.89)**
Sex					
Female	243,300	228	243,300	302	**0.75 (0.63–0.89)**
Male	180,833	154	180,833	186	0.81 (0.66–1.01)
BMI					
< 18.5	2700	10	2700	10	0.42 (0.11–1.62)
18.5–24.9	54,235	48	54,235	62	0.80 (0.55–1.17)
25–29.9	84,919	88	84,919	101	0.87 (0.65–1.15)
≥ 30	114,982	112	114,982	135	0.83 (0.64–1.06)
Ethnicity					
Not Hispanic or Latino	318,930	296	318,930	360	**0.82 (0.70–0.95)**
Hispanic or Latino	26,493	31	26,493	28	1.10 (0.66–1.83)
Race					
White	320,886	270	320,886	331	**0.81 (0.69–0.95)**
Black or African American	43,372	65	43,372	95	**0.66 (0.48–0.91)**
Asian	24,178	12	24,178	20	0.61 (0.30–1.26)
Native Hawaiian or other Pacific Islander	3103	10	3103	10	0.94 (0.06–14.95)
American Indian or Alaska Native	1678	10	1678	0	N/A
Other race	11,889	13	11,889	18	0.68 (0.33–1.39)
Comorbidities					
Hypertensive diseases	177,009	199	177,009	240	0.83 (0.69–1.01)
Diabetes mellitus	77,186	89	77,186	132	**0.68 (0.52–0.89)**
Ischemic heart diseases	48,328	60	48,328	76	0.81 (0.58–1.14)
Hyperlipidemia	179,955	188	179,955	241	**0.78 (0.65–0.95)**
Heart failure	23,125	20	23,125	38	**0.52 (0.30–0.90)**
Cerebral infarction	7313	11	7313	11	0.97 (0.42–2.24)

*Note:* If the patient's count is 1–10, the results indicate a count of 10. Bold values are statistical significance.

Abbreviation: N/A, not applicable.

**Table 4:** Risk of uveitis exposed to antiviral compared to non‐antiviral.

	Antiviral		Non‐antiviral		
	*N*	No. of event	*N*	No. of event	HR (95% CI)
COVID‐19 vaccine					
No	360,793	293	360,793	346	**0.84 (0.72–0.98)**
Yes	74,725	95	74,725	132	**0.75 (0.57–0.98)**
Breakthrough^a^	2154	10	2154	10	1.47 (0.25–8.82)
Ritonavir‐nirmatrelvir (Paxlovid)	344,109	313	344,109	366	**0.83 (0.71–0.96)**
Molnupiravir	38,496	33	38,496	35	0.90 (0.56–1.44)
Remdesivir	60,232	48	60,232	71	0.80 (0.56–1.16)
CEV1 (1 year prior to the index date)	3635	11	3635	10	2.20 (0.77–6.34)
CEV2 (1 year prior to the index date)	51,070	62	51,070	64	0.98 (0.69–1.39)
CEV3 (1 year prior to the index date)	146,971	183	146,971	207	0.89 (0.73–1.08)
CEV1 (no time restriction prior to the index date)	6022	16	6022	10	1.76 (0.78–3.99)
CEV2 (no time restriction prior to the index date)	92,972	110	92,972	137	0.82 (0.63–1.05)
CEV3 (no time restriction prior to the index date)	245,317	293	245,317	310	0.95 (0.81–1.12)
Inclusion period = 2022/01–2022/12	186,900	194	186,900	284	**0.69 (0.57–0.82)**
Inclusion period = 2023/01–2023/12	150,238	149	150,238	147	0.95 (0.75–1.19)
Inclusion period = 2024/01–2024/12	102,976	50	102,976	48	0.97 (0.65–1.44)

*Note:* If the patient's count is 1–10, the results indicate a count of 10. Bold values are statistical significance.

Abbreviation: N/A, not applicable.

^a^ Breakthrough: Defined as a COVID‐19 diagnosis within 14 days after completion of COVID‐19 vaccination [4, 5, 6, 7].

Stratification by race showed a significantly lower risk in White (HR = 0.81; 95% CI: 0.69–0.95) and Black or African American patients (HR = 0.66; 95% CI: 0.48–0.91), while the trend was not significant for other racial groups or across any BMI strata.

Among patients with comorbidities, the protective effect was significant for those with diabetes mellitus (HR = 0.68; 95% CI: 0.52–0.89), hyperlipidemia (HR = 0.78; 95% CI: 0.65–0.95), or heart failure (HR = 0.52; 95% CI: 0.30–0.90). When analyzed by specific antiviral agent, only ritonavir‐nirmatrelvir (Paxlovid) was associated with a statistically significant risk reduction (HR = 0.83; 95% CI: 0.71–0.96).

The benefit of antiviral therapy was consistent regardless of COVID‐19 vaccination history, with significant risk reductions in both vaccinated (HR = 0.75; 95% CI: 0.57–0.98) and unvaccinated individuals (HR = 0.84; 95% CI: 0.72–0.98). In contrast, no statistically significant association was detected among patients with breakthrough infections, defined as SARS‐CoV‐2 infection occurring within 14 days of vaccination (HR = 1.47; 95% CI: 0.25–8.82). However, this subgroup was small, with a limited number of uveitis events, resulting in wide CIs and insufficient statistical power to draw firm conclusions. Interestingly, when stratified by the year of diagnosis, the protective effect was significant only for patients included in 2022 (HR = 0.69; 95% CI: 0.57–0.82), with no significant association observed for those diagnosed in 2023 or 2024. In contrast, analyses based on CEV classifications showed no statistically significant differences in risk.

## 4 Discussion

### 4.1 Novel Findings

Using large‐scale real‐world data from the TriNetX platform, we compared 438,455 adult COVID‐19 patients who received early antiviral therapy (Paxlovid, molnupiravir, or remdesivir) within 5 days of diagnosis with an equal number of matched untreated controls. Early antiviral use was associated with a significantly lower risk of new‐onset uveitis (HR = 0.81), with the strongest protective effect observed within the first 3 months after infection (HR = 0.62). Subtype analyses showed a significant reduction in iridocyclitis, and subgroup analyses demonstrated that the association was generally consistent across strata defined by age, sex, race, and comorbidity burden. To our knowledge, this represents one of the largest studies to date evaluating the relationship between SARS‐CoV‐2 infection, early antiviral treatment, and subsequent development of uveitis.

### 4.2 Clinical Implications and Comparisons to Literature

Numerous prior reports have documented cases of iridocyclitis following SARS‐CoV‐2 infection, and viral RNA has been detected in ocular secretions. Although proposed mechanisms vary, the causal link is well established. In Al‐Namaeh's review of COVID‐19 ocular complications, among 101 case reports and series, four specifically mentioned uveitis (Benito‐Pascual et al.; Sanjay, Gowda, et al.; Alonso et al.; Sanjay, Srinivasan, et al.) [6, 14, 15, 16, 17]. Feng et al. [7] described 18 cases of post‐COVID uveitis. In our own 2023 analysis, COVID‐19 patients exhibited a significantly increased risk of developing uveitic diseases compared with non‐infected controls, with an HR of 1.18 (95% CI: 1.03–1.34) sustained through 24 months [18].

Paxlovid, molnupiravir, and remdesivir each effectively inhibit viral replication, reducing both systemic and ocular viral loads and attenuating the local inflammatory cascades triggered by direct uveal invasion [19]. By including over 400,000 matched patients and extending follow‐up to 3 years, our study provides robust evidence that early antiviral therapy may mitigate ocular immune‐mediated complications. Given these findings, future COVID‐19 guidelines should explicitly recommend antiviral use in clinical care pathways, especially for patients at high risk for uveitis.

We observed that the strongest protective association occurred within the first 3 months after infection, a time frame that aligns with prior reports indicating that the peak risk of uveitis among COVID‐19 patients occurs approximately 2 to 6 months after diagnosis [18]. This temporal concordance supports a hypothesized model in which molecular mimicry between viral antigens and intraocular self‐antigens may initiate an autoreactive immune response [20, 21]. By reducing viral load early in the course of infection, antiviral therapy may attenuate or prevent this downstream inflammatory cascade. In addition, IgM and IgG autoantibody titers are known to peak within 2 to 3 weeks after infection, and the subsequent rise in uveitis incidence at 2 to 3 months may therefore correspond to the window during which early antiviral intervention confers the greatest protective effect [20].

In age‐stratified analyses, both patients aged ≥ 65 years (HR = 0.74) and those aged 18–64 (HR = 0.82) experienced significant risk reductions. Interestingly, non‐infectious uveitis incidence traditionally increases with age, yet COVID‐19‐associated uveitis risk appears similar in younger and older adults [18, 22, 23]. This suggests that age may not be a primary modifier of SARS‐CoV‐2‐related uveitis risk or antiviral efficacy in this context.

In sex‐stratified analyses, females appeared to derive a greater protective association from antiviral therapy (HR = 0.75) than males (HR = 0.83), although the estimate in males did not reach statistical significance. Epidemiologically, women have a higher incidence of uveitis, which has been linked to a greater prevalence of autoimmune diseases and to hormonal influences, including estrogen. Experimental models suggest that uveitis is largely mediated by Th1 and Th17 immune responses, pathways that may be modulated by sex hormones [24, 25, 26]. It is therefore plausible that post‐COVID‐19 uveitis reflects an autoimmune process triggered by tissue injury and self‐antigen release, with early viral suppression conferring greater relative benefit in females. By contrast, analyses stratified by BMI and race did not demonstrate statistically significant differences, which may reflect limited sample sizes among non‐White individuals and patients at extreme BMI ranges within TriNetX.

We further examined outcomes in CEV subgroups as defined by the UK Health Security Agency, including CEV1 with severe immunosuppression, CEV2 with moderate immunosuppression, and CEV3 comprising patients at high risk without immunosuppression [27]. In the CEV1 subgroup, sample sizes were insufficient to detect meaningful differences. In both CEV2 and CEV3, antiviral use was associated with a lower incidence of uveitis compared with untreated CEV patients, although the magnitude of risk reduction was smaller than that observed in the non‐CEV cohort. This attenuation may reflect differences in underlying pathophysiology. Post COVID‐19 uveitis is thought to be driven in part by immune hyperactivation, whereas CEV patients frequently receive immunomodulatory therapies and may therefore exhibit a relatively blunted inflammatory response, which could limit the extent to which antivirals reduce subsequent immune‐mediated uveal inflammation.

We stratified analyses by calendar year to approximate the impact of different SARS‐CoV‐2 variants, using diagnosis date as a proxy because direct variant‐level data were unavailable. To align with the clinical availability of oral antivirals, analyses were restricted to diagnoses from 2022 onward. In 2022, when Omicron sublineages predominated, we observed the only statistically significant reduction in uveitis risk [28]. In 2023, a period dominated by XBB.1.5 and JN.1, which together accounted for nearly 90% of U.S. sequences, point estimates continued to favor antiviral use but did not reach statistical significance [29, 30]. Similar nonsignificant but directionally consistent associations were observed in 2024, when multiple KP‐series variants emerged [31]. These findings should be interpreted cautiously, as calendar year is an imperfect surrogate for the infecting variant, and substantial within‐year heterogeneity is likely. Nevertheless, the consistency of point estimates suggests a potential benefit of early antiviral therapy across successive Omicron‐era variants.

Differential associations were observed across commonly used COVID‐19 antivirals. These included oral Paxlovid (nirmatrelvir/ritonavir), intravenous remdesivir, and oral molnupiravir, which act through distinct antiviral mechanisms [32]. Paxlovid was the most frequently prescribed and demonstrated the strongest protective signal against uveitis in our cohort, whereas remdesivir and molnupiravir showed only non‐significant trends toward benefit, which is likely because far more patients received Paxlovid [33]. With smaller sample sizes, the other two drugs did not show significant differences. The observed protective association may reflect differences in pharmacologic and antiviral properties among agents, although these explanations remain speculative. Paxlovid is a small‐molecule antiviral with relatively low plasma protein binding, which could theoretically facilitate distribution into ocular compartments such as tears or the aqueous humor; however, direct pharmacokinetic data in ocular tissues are currently lacking. In addition, Paxlovid inhibits SARS‐CoV‐2 replication by blocking viral polyprotein cleavage, a mechanism that may allow more rapid suppression of viral replication and early inflammatory signaling. By contrast, remdesivir and molnupiravir target viral RNA replication through polymerase inhibition and lethal mutagenesis, respectively, and their antiviral effects may accrue more gradually. If post‐COVID‐19 uveitis is triggered in part by early immune activation and subsequent autoantibody formation, earlier viral suppression could plausibly confer greater protection. This hypothesis requires further mechanistic investigation but may partially explain the findings observed in our study [34, 35]. Finally, we found that vaccination status did not alter the association. To elaborate, antiviral therapy reduced the risk of post‐COVID uveitis regardless of mRNA COVID‐19 vaccination status. Although the potential for mRNA vaccines to induce ocular adverse events remains an ongoing investigation, our findings suggest that prior vaccination does not attenuate the apparent ocular protective effect of early antiviral treatment.

### 4.3 Strengths and Limitations

This study is one of the largest investigations to date of post‐COVID‐19 uveitis risk and the impact of antiviral therapy. It also includes extensive subgroup analyses stratified by age, sex, race, comorbidity burden, vaccination status, CEV tier, diagnosis year as a proxy for dominant circulating variants, and individual antiviral agents, thereby providing insight into treatment effects across diverse patient populations and viral contexts.

Nevertheless, this study has several limitations. First, the source population was confined to the U.S. healthcare system, in which White patients remain overrepresented, potentially limiting generalizability. Second, the database does not capture vaccine manufacturer, dosing regimen, or timing, precluding analyses of specific vaccine–antiviral interactions. Although antiviral treatment was assumed to be initiated within 5 days of diagnosis, information on actual adherence or completion of treatment courses was unavailable because the data were de‐identified and aggregated rather than individualized. Third, analyses stratified by SARS‐CoV‐2 variant relied on calendar year as a proxy for dominant circulating strains, as individual‐level variant data were unavailable, introducing potential misclassification due to within‐year heterogeneity. Fourth, although many subgroup analyses demonstrated directionally consistent trends, sample sizes were insufficient to support statistically robust comparisons in several categories, including BMI strata, CEV1 patients, individuals with breakthrough infections, and recipients of antivirals other than Paxlovid. Finally, certain drug‐related factors could not be fully accounted for. For example, remdesivir is administered intravenously and typically reflects inpatient use, molnupiravir is contraindicated in pregnancy, and Paxlovid is a CYP3A inhibitor with numerous potential drug–drug interactions. Owing to limitations in sample size and study scope, we were unable to adjust for all of these considerations comprehensively.

## 5 Conclusion

Early antiviral treatment for COVID‐19 was associated with a lower risk of subsequent uveitis, with the strongest associations observed within 3 months of infection, among Paxlovid recipients, in female patients, and during the 2022 Omicron‐dominant period. These findings suggest that antiviral therapy may offer potential ocular benefits in addition to reducing systemic disease severity, particularly in individuals at higher risk of post–COVID‐19 inflammatory complications.

## Author Contributions

Conceptualization: Chun‐Ju Lin and Hou‐Ting Kuo. Methodology: Hou‐Ting Kuo, Alan Y. Hsu, Chun‐Ju Lin, and Huan‐Sheng Chen. Formal Analysis: Yu‐Hsun Wang and Hou‐Ting Kuo. Investigation: De‐Yi Liu, Bing‐Qi Wu, James Cheng‐Chung Wei, Ning‐Yi Hsia, Yi‐Ching Shao, Chun‐Ting Lai, Chun‐Chi Chiang, Hsin Tseng, Ho‐Che Hsu, and Yi‐Yu Tsai. Writing – original draft: Hou‐Ting Kuo and Alan Y. Hsu. Writing – review and editing: All authors. Supervision: Chun‐Ju Lin and Huan‐Sheng Chen. All authors have read and agreed to the published version of the manuscript.

## Policy on Using ChatGPT and Similar AI Tools

No artificial intelligence (AI) tools or software were used in the writing, analysis, or preparation of this manuscript.

## Funding

The authors have nothing to report.

## Conflicts of Interest

The authors declare no conflicts of interest.

## Supporting information

Supporting File

## Data Availability

All relevant data have been included in this study. Further requests for data generated from this study are available from the corresponding author upon reasonable request. Additionally, requests for data can be sent by logging on to the TriNetX platform (https://live.trinetx.com/).
