# Supplementary material for: Impact of Early COVID‐19 Antiviral Therapy on the Incidence of Uveitis: A Retrospective Cohort Study Using the TriNetX Database
Source: Immun Inflamm Dis. 2026 May 6;14(5):e70455. doi: 10.1002/iid3.70455 (PMC13149768; doi:10.1002/iid3.70455)
Supplement: Supplementary file 1 — Supporting File [file IID3-14-e70455-s001.docx]

**Supplementary Table 1** Coding definitions for inclusion, exclusion, and uveitis

|  | Code |
| --- | --- |
| **COVID-19** |  |
| SARS coronavirus 2 and related RNA [Presence]  (labResult: Positive) | TNX:9088 |
| SARS coronavirus 2 IgG IgM Ab [Presence] in Serum or  Plasma (labResult: Positive) | TNX:9089 |
| SARS-CoV-2 (COVID-19) IgG Ab [Units/volume] in  Serum or Plasma by Immunoassay (at least 0.10  [arb'U]/mL) | LNC:94505-5 |
| SARS-CoV-2 (COVID-19) IgM Ab [Units/volume] in  Serum or Plasma by Immunoassay (at least 0.10  [arb'U]/mL) | LNC:94506-3 |
| SARS-CoV-2 (COVID-19) IgA Ab [Presence] in Serum or  Plasma by Immunoassay (labResult: Positive) | LNC:94562-6 |
| SARS-CoV-2 (COVID-19) Ab [Presence] in Serum or  Plasma by Immunoassay (labResult: Positive) | LNC:94762-2 |
| SARS-CoV-2 (COVID-19) Ab [Units/volume] in Serum  or Plasma by Immunoassay (at least 0.10 [IU]/mL) | LNC:94769-7 |
| SARS-CoV-2 (COVID-19) Ag [Presence] in Respiratory  specimen by Rapid immunoassay (labResult: Positive) | LNC:94558-4 |
| SARS-CoV+SARS-CoV-2 (COVID-19) Ag [Presence] in  Respiratory specimen by Rapid immunoassay (labResult:  Positive) | LNC:95209-3 |
| SARS-CoV-2 (COVID-19) Ag [Presence] in Upper  respiratory specimen by Immunoassay (labResult:  Positive) | LNC:96119-3 |
| COVID-19 | ICD-10-CM:U07.1 |
| **COVID-19 antiviral medications** |  |
| Paxlovid |  |
| Ritonavir | RxNorm:85762 |
| Nirmatrelvir | RxNorm:2587892 |
| Molnupiravir | RxNorm:2587901 |
| Remdesivir |  |
| Remdesivir | RxNorm:2284718 |
| Injection, remdesivir, 1 mg | HCPCS:J0248 |
| **Exclude previous underlying disease** |  |
| Viral hepatitis | ICD-10-CM:B15-B19 |
| Human immunodeficiency virus | ICD-10-CM:B20, R75, Z21, B97.35 |
| Tuberculosis | ICD-10-CM:A15-A19 |
| Syphilis | ICD-10-CM:A50-A53 |
| Rheumatoid arthritis | ICD-10-CM:M05-M06 |
| Systemic lupus erythematosus | ICD-10-CM:M32 |
| Sjogren's syndrome | ICD-10-CM:M35.0 |
| Rheumatic disease | ICD-10-CM:M31.6, M33.03, M33.13, M33.2, M33.90, M33.93, M34.0, M34.1, M34.9 |
| Behcet's disease | ICD-10-CM:M35.2 |
| Meniere's disease | ICD-10-CM:H81.0 |
| Sarcoidosis | ICD-10-CM:D86 |
| Susac syndrome | ICD-10-CM:I67.7 |
| **Other anti-viral drugs** |  |
| Cyclic amines | ATC:J05AC |
| Antivirals | ATC:D06BB |
| Phosphonic acid derivatives | ATC:J05AD |
| Nucleoside and nucleotide reverse transcriptase inhibitors | ATC:J05AF |
| Non-nucleoside reverse transcriptase inhibitors | ATC:J05AG |
| Neuraminidase inhibitors | ATC:J05AH |
| Integrase inhibitors | ATC:J05AJ |
| Antivirals for treatment of HCV infections | ATC:J05AP |
| Antivirals for treatment of HIV infections, combinations | ATC:J05AR |
| Other antivirals | ATC:J05AX |
| Vidarabine | RxNorm:11194 |
| Brincidofovir | RxNorm:2599518 |
| Valganciclovir | RxNorm:275891 |
| Acyclovir | RxNorm:281 |
| Ganciclovir | RxNorm:4678 |
| Idoxuridine | RxNorm:5653 |
| Penciclovir | RxNorm:59839 |
| Famciclovir | RxNorm:68099 |
| Valacyclovir | RxNorm:73645 |
| Cidofovir | RxNorm:83171 |
| Indinavir | RxNorm:114289 |
| Nelfinavir | RxNorm:134527 |
| Tipranavir | RxNorm:190548 |
| Amprenavir | RxNorm:228656 |
| Atazanavir | RxNorm:343047 |
| Fosamprenavir | RxNorm:358262 |
| Darunavir | RxNorm:460132 |
| Saquinavir | RxNorm:83395 |
| **Uveitis** |  |
| Iridocyclitis | ICD-10-CM:H20 |
| Focal chorioretinal inflammation | ICD-10-CM:H30.0 |
| Unspecified disseminated chorioretinal inflammation | ICD-10-CM:H30.109 |
| Posterior cyclitis | ICD-10-CM:H30.2 |
| Retinal Vasculitis | ICD-10-CM:H35.06 |
| Panuveitis | ICD-10-CM:H44.11 |

TNX:TrinetX curated.

LNC:Logical Observation Identifier Name and Codes, LOINC.

ICD-10-CM: International Classification of Diseases, Tenth Revision, Clinical Modification.

RxNorm:Medical prescription normalized Medical prescription.

HCPCS: Healthcare Common Procedure Coding System.

ATC:Anatomical Therapeutic Chemical.

**Supplementary Table 2** Coding definitions for covariates

|  | ICD-10-CM |
| --- | --- |
| **Comorbidities** |  |
| Hypertensive diseases | ICD-10-CM:I10-I1A |
| Diabetes mellitus | ICD-10-CM:E08-E13 |
| Ischemic heart diseases | ICD-10-CM:I20-I25 |
| Disorders of lipoprotein metabolism and other lipidemias | ICD-10-CM:E78 |
| Heart failure | ICD-10-CM:I50 |
| Cerebral infarction | ICD-10-CM:I63 |
| **Medications** |  |
| Ophthalmologicals | ATC:S01 |
| Corticosteroids for systemic use | ATC:H02 |
| Acetaminophen | RxNorm:161 |
| Antiinflammatory and antirheumatic products, non-steroids | ATC:M01A |
| Aspirin | RxNorm:1191 |
| Metformin | RxNorm:6809 |
| Bevacizumab | RxNorm:253337 |
| Interferon beta-1a | RxNorm:75917 |
| Fingolimod | RxNorm:1012892 |
| Tocilizumab | RxNorm:612865 |
| Sarilumab | RxNorm:1923319 |

ICD-10-CM: International Classification of Diseases, Tenth Revision, Clinical Modification.

RxNorm:Medical prescription normalized Medical prescription.

ATC:Anatomical Therapeutic Chemical.

**Supplementary Table 3** Coding definitions for COVID-19 vaccine and CEV1-3

|  | Code |
| --- | --- |
| **COVID-19 vaccine** |  |
| Severe acute respiratory syndrome coronavirus 2 (SARS-CoV-2) (coronavirus disease [COVID-19]) vaccine, mRNA-LNP, spike protein, preservative free, 30 mcg/0.3 mL dosage, diluent reconstituted, for intramuscular use | CPT:91300 |
| Severe acute respiratory syndrome coronavirus 2 (SARS-CoV-2) (coronavirus disease [COVID-19]) vaccine, mRNA-LNP, spike protein, preservative free, 100 mcg/0.5 mL dosage, for intramuscular use | CPT:91301 |
| Severe acute respiratory syndrome coronavirus 2 (SARS-CoV-2) (coronavirus disease [COVID-19]) vaccine, DNA, spike protein, chimpanzee adenovirus Oxford 1 (ChAdOx1) vector, preservative free, 5x1010 viral particles/0.5 mL dosage, for intramuscular use | CPT:91302 |
| Severe acute respiratory syndrome coronavirus 2 (SARS-CoV-2) (coronavirus disease [COVID-19]) vaccine, DNA, spike protein, adenovirus type 26 (Ad26) vector, preservative free, 5x1010 viral particles/0.5 mL dosage, for intramuscular use | CPT:91303 |
| Severe acute respiratory syndrome coronavirus 2 (SARS-CoV-2) (coronavirus disease [COVID-19]) vaccine, recombinant spike protein nanoparticle, saponin-based adjuvant, 5 mcg/0.5 mL dosage, for intramuscular use | CPT:91304 |
| Severe acute respiratory syndrome coronavirus 2 (SARS-CoV-2) (coronavirus disease [COVID-19]) vaccine, mRNA-LNP, spike protein, preservative free, 30 mcg/0.3 mL dosage, tris-sucrose formulation, for intramuscular use | CPT:91305 |
| Severe acute respiratory syndrome coronavirus 2 (SARS-CoV-2) (coronavirus disease [COVID-19]) vaccine, mRNA-LNP, spike protein, preservative free, 50 mcg/0.25 mL dosage, for intramuscular use | CPT:91306 |
| Severe acute respiratory syndrome coronavirus 2 (SARS-CoV-2) (coronavirus disease [COVID-19]) vaccine, mRNA-LNP, spike protein, preservative free, 10 mcg/0.2 mL dosage, diluent reconstituted, tris-sucrose formulation, for intramuscular use (deprecated 2024) | CPT:91307 |
| Severe acute respiratory syndrome coronavirus 2 (SARS-CoV-2) (coronavirus disease [COVID-19]) vaccine, mRNA-LNP, spike protein, preservative free, 3 mcg/0.2 mL dosage, diluent reconstituted, tris-sucrose formulation, for intramuscular use (deprecated 2024) | CPT:91308 |
| Severe acute respiratory syndrome coronavirus 2 (SARS-CoV-2) (coronavirus disease [COVID-19]) vaccine, mRNA-LNP, spike protein, preservative free, 50 mcg/0.5 mL dosage, for intramuscular use (deprecated 2024) | CPT:91309 |
| Severe acute respiratory syndrome coronavirus 2 (SARS-CoV-2) (coronavirus disease [COVID-19]) vaccine, mRNA-LNP, spike protein, preservative free, 25 mcg/0.25 mL dosage, for intramuscular use (deprecated 2024) | CPT:91311 |
| Severe acute respiratory syndrome coronavirus 2 (SARS-CoV-2) (coronavirus disease [COVID-19]) vaccine, mRNA-LNP, spike protein, 3 mcg/0.3 mL dosage, tris-sucrose formulation, for intramuscular use | CPT:91318 |
| Severe acute respiratory syndrome coronavirus 2 (SARS-CoV-2) (coronavirus disease [COVID-19]) vaccine, mRNA-LNP, spike protein, 10 mcg/0.3 mL dosage, tris-sucrose formulation, for intramuscular use | CPT:91319 |
| Severe acute respiratory syndrome coronavirus 2 (SARS-CoV-2) (coronavirus disease [COVID-19]) vaccine, mRNA-LNP, spike protein, 30 mcg/0.3 mL dosage, tris-sucrose formulation, for intramuscular use | CPT:91320 |
| Severe acute respiratory syndrome coronavirus 2 (SARS-CoV-2) (coronavirus disease [COVID-19]) vaccine, mRNA-LNP, 25 mcg/0.25 mL dosage, for intramuscular use | CPT:91321 |
| Severe acute respiratory syndrome coronavirus 2 (SARS-CoV-2) (coronavirus disease [COVID-19]) vaccine, mRNA-LNP, 50 mcg/0.5 mL dosage, for intramuscular use | CPT:91322 |
| Immunization administration by intramuscular injection of severe acute respiratory syndrome coronavirus 2 (SARS-CoV-2) (coronavirus disease [COVID-19]) vaccine, mRNA-LNP, spike protein, preservative free, 30 mcg/0.3 mL dosage, diluent reconstituted; first dose (deprecated 2024) | CPT:0001A |
| Immunization administration by intramuscular injection of severe acute respiratory syndrome coronavirus 2 (SARS-CoV-2) (coronavirus disease [COVID-19]) vaccine, mRNA-LNP, spike protein, preservative free, 30 mcg/0.3 mL dosage, diluent reconstituted; second dose (deprecated 2024) | CPT:0002A |
| Immunization administration by intramuscular injection of severe acute respiratory syndrome coronavirus 2 (SARS-CoV-2) (coronavirus disease [COVID-19]) vaccine, mRNA-LNP, spike protein, preservative free, 30 mcg/0.3 mL dosage, diluent reconstituted; third dose (deprecated 2024) | CPT:0003A |
| Immunization administration by intramuscular injection of severe acute respiratory syndrome coronavirus 2 (SARS-CoV-2) (coronavirus disease [COVID-19]) vaccine, mRNA-LNP, spike protein, preservative free, 30 mcg/0.3 mL dosage, diluent reconstituted; booster dose (deprecated 2024) | CPT:0004A |
| Immunization administration by intramuscular injection of severe acute respiratory syndrome coronavirus 2 (SARS-CoV-2) (coronavirus disease [COVID-19]) vaccine, mRNA-LNP, spike protein, preservative free, 100 mcg/0.5 mL dosage; first dose (deprecated 2024) | CPT:0011A |
| Immunization administration by intramuscular injection of severe acute respiratory syndrome coronavirus 2 (SARS-CoV-2) (coronavirus disease [COVID-19]) vaccine, mRNA-LNP, spike protein, preservative free, 100 mcg/0.5 mL dosage; second dose (deprecated 2024) | CPT:0012A |
| Immunization administration by intramuscular injection of severe acute respiratory syndrome coronavirus 2 (SARS-CoV-2) (coronavirus disease [COVID-19]) vaccine, mRNA-LNP, spike protein, preservative free, 100 mcg/0.5 mL dosage; third dose (deprecated 2024) | CPT:0013A |
| Immunization administration by intramuscular injection of severe acute respiratory syndrome coronavirus 2 (SARS-CoV-2) (coronavirus disease [COVID-19]) vaccine, DNA, spike protein, adenovirus type 26 (Ad26) vector, preservative free, 5x1010 viral particles/0.5 mL dosage; single dose (deprecated 2024) | CPT:0031A |
| Immunization administration by intramuscular injection of severe acute respiratory syndrome coronavirus 2 (SARS-CoV-2) (coronavirus disease [COVID-19]) vaccine, recombinant spike protein nanoparticle, saponin-based adjuvant, preservative free, 5 mcg/0.5 mL dosage; first dose (deprecated 2024) | CPT:0041A |
| Immunization administration by intramuscular injection of severe acute respiratory syndrome coronavirus 2 (SARS-CoV-2) (coronavirus disease [COVID-19]) vaccine, recombinant spike protein nanoparticle, saponin-based adjuvant, preservative free, 5 mcg/0.5 mL dosage; second dose (deprecated 2024) | CPT:0042A |
| Immunization administration by intramuscular injection of severe acute respiratory syndrome coronavirus 2 (SARS-CoV-2) (coronavirus disease [COVID-19]) vaccine, mRNA-LNP, spike protein, preservative free, 50 mcg/0.25 mL dosage, booster dose (deprecated 2024) | CPT:0064A |
| Immunization administration by intramuscular injection of severe acute respiratory syndrome coronavirus 2 (SARS-CoV-2) (coronavirus disease [COVID-19]) vaccine, mRNA-LNP, spike protein, preservative free, 10 mcg/0.2 mL dosage, diluent reconstituted, tris-sucrose formulation; first dose (deprecated 2024) | CPT:0071A |
| Immunization administration by intramuscular injection of severe acute respiratory syndrome coronavirus 2 (SARS-CoV-2) (coronavirus disease [COVID-19]) vaccine, mRNA-LNP, spike protein, preservative free, 10 mcg/0.2 mL dosage, diluent reconstituted, tris-sucrose formulation; second dose (deprecated 2024) | CPT:0072A |
| Immunization administration by intramuscular injection of severe acute respiratory syndrome coronavirus 2 (SARS-CoV-2) (coronavirus disease [COVID-19]) vaccine, mRNA-LNP, spike protein, preservative free, 10 mcg/0.2 mL dosage, diluent reconstituted, tris-sucrose formulation; third dose (deprecated 2024) | CPT:0073A |
| Immunization administration by intramuscular injection of severe acute respiratory syndrome coronavirus 2 (SARS-CoV-2) (coronavirus disease [COVID-19]) vaccine, mRNA-LNP, spike protein, preservative free, 10 mcg/0.2 mL dosage, diluent reconstituted, tris-sucrose formulation; booster dose (deprecated 2024) | CPT:0074A |
| Immunization administration by intramuscular injection of severe acute respiratory syndrome coronavirus 2 (SARS-CoV-2) (coronavirus disease [COVID-19]) vaccine, mRNA-LNP, spike protein, preservative free, 3 mcg/0.2 mL dosage, diluent reconstituted, tris-sucrose formulation; first dose (deprecated 2024) | CPT:0081A |
| Immunization administration by intramuscular injection of severe acute respiratory syndrome coronavirus 2 (SARS-CoV-2) (coronavirus disease [COVID-19]) vaccine, mRNA-LNP, spike protein, preservative free, 3 mcg/0.2 mL dosage, diluent reconstituted, tris-sucrose formulation; second dose (deprecated 2024) | CPT:0082A |
| Immunization administration by intramuscular injection of severe acute respiratory syndrome coronavirus 2 (SARS-CoV-2) (coronavirus disease [COVID-19]) vaccine, mRNA-LNP, spike protein, preservative free, 3 mcg/0.2 mL dosage, diluent reconstituted, tris-sucrose formulation; third dose (deprecated 2024) | CPT:0083A |
| Immunization administration by intramuscular injection of severe acute respiratory syndrome coronavirus 2 (SARS-CoV-2) (coronavirus disease [COVID-19]) vaccine, mRNA-LNP, spike protein, preservative free, 50 mcg/0.5 mL dosage; first dose, when administered to individuals 6 through 11 years (deprecated 2024) | CPT:0091A |
| Immunization administration by intramuscular injection of severe acute respiratory syndrome coronavirus 2 (SARS-CoV-2) (coronavirus disease [COVID-19]) vaccine, mRNA-LNP, spike protein, preservative free, 50 mcg/0.5 mL dosage; second dose, when administered to individuals 6 through 11 years (deprecated 2024) | CPT:0092A |
| Immunization administration by intramuscular injection of severe acute respiratory syndrome coronavirus 2 (SARS-CoV-2) (coronavirus disease [COVID-19]) vaccine, mRNA-LNP, spike protein, preservative free, 50 mcg/0.5 mL dosage; third dose, when administered to individuals 6 through 11 years (deprecated 2024) | CPT:0093A |
| Immunization administration by intramuscular injection of severe acute respiratory syndrome coronavirus 2 (SARS-CoV-2) (coronavirus disease [COVID-19]) vaccine, mRNA-LNP, spike protein, preservative free, 50 mcg/0.5 mL dosage; booster dose, when administered to individuals 18 years and over (deprecated 2024) | CPT:0094A |
| Immunization administration by intramuscular injection of severe acute respiratory syndrome coronavirus 2 (SARS-CoV-2) (coronavirus disease [COVID-19]) vaccine, mRNA-LNP, spike protein, preservative free, 25 mcg/0.25 mL dosage; first dose (deprecated 2024) | CPT:0111A |
| Immunization administration by intramuscular injection of severe acute respiratory syndrome coronavirus 2 (SARS-CoV-2) (coronavirus disease [COVID-19]) vaccine, mRNA-LNP, spike protein, preservative free, 25 mcg/0.25 mL dosage; second dose (deprecated 2024) | CPT:0112A |
| Immunization administration by intramuscular injection of severe acute respiratory syndrome coronavirus 2 (SARS-CoV-2) (coronavirus disease [COVID-19]) vaccine, mRNA-LNP, spike protein, preservative free, 25 mcg/0.25 mL dosage; third dose (deprecated 2024) | CPT:0113A |
| SARS-CoV-2 (COVID-19) Vaccine | CVX:213 |
| SARS-CoV-2 (COVID-19) vaccine, mRNA spike protein | RxNorm:2468231 |
| SARS-COV-2 (COVID-19) vaccine, vector non-replicating | RxNorm:2479831 |
| **CEV1** |  |
| Transplanted organ and tissue status | ICD-10-CM:Z94 |
| Other and unspecified malignant neoplasms of lymphoid, hematopoietic and related tissue | ICD-10-CM:C96 |
| Rituximab | RxNorm:121191 |
| Ocrelizumab | RxNorm:1876366 |
| Ofatumumab | RxNorm:712566 |
| Obinutuzumab | RxNorm:974779 |
| Ibritumomab tiuxetan | RxNorm:262323 |
| Inebilizumab | RxNorm:2373951 |
| Combined immunodeficiencies | ICD-10-CM:D81 |
| Immunodeficiency associated with other major defects | ICD-10-CM:D82 |
| **CEV2** |  |
| Encounter for other aftercare and medical care | ICD-10-CM:Z51 |
| Immunodeficiency with predominantly antibody defects | ICD-10-CM:D80 |
| Other disorders involving the immune mechanism, not elsewhere classified | ICD-10-CM:D89 |
| Chronic kidney disease | ICD-10-CM:N18 |
| Dependence on renal dialysis | ICD-10-CM:Z99.2 |
| Unspecified nephritic syndrome with unspecified morphologic changes | ICD-10-CM:N05.9 |
| Long term (current) use of systemic steroids | ICD-10-CM:Z79.52 |
| **CEV3** |  |
| Cystic fibrosis | ICD-10-CM:E84 |
| Exocrine pancreatic insufficiency | ICD-10-CM:K86.81 |
| Pulmonary hypertension, unspecified | ICD-10-CM:I27.20 |
| Other interstitial pulmonary diseases | ICD-10-CM:J84 |
| Other chronic obstructive pulmonary disease | ICD-10-CM:J44 |
| Asthma | ICD-10-CM:J45 |
| Sickle-cell disorders | ICD-10-CM:D57 |
| Hemolytic-uremic syndrome | ICD-10-CM:D59.3 |
| Maple-syrup-urine disease | ICD-10-CM:E71.0 |
| Other disorders of branched-chain amino-acid metabolism | ICD-10-CM:E71.1 |
| Disorders of urea cycle metabolism | ICD-10-CM:E72.2 |
| Disorders of lysine and hydroxylysine metabolism | ICD-10-CM:E72.3 |
| Long term (current) use of insulin | ICD-10-CM:Z79.4 |
| Hyposplenism | ICD-10-CM:D73.0 |
| Neoplasms | ICD-10-CM:C00-D49 |
| Down syndrome | ICD-10-CM:Q90 |
| Cerebral palsy | ICD-10-CM:G80 |
| Intellectual Disabilities | ICD-10-CM:F70-F79 |
| Nonrheumatic mitral valve disorders | ICD-10-CM:I34 |
| Nonrheumatic aortic valve disorders | ICD-10-CM:I35 |
| Nonrheumatic tricuspid valve disorders | ICD-10-CM:I36 |
| Nonrheumatic pulmonary valve disorders | ICD-10-CM:I37 |
| Dependence on respirator [ventilator] status | ICD-10-CM:Z99.11 |
| Encounter for respirator [ventilator] dependence during power failure | ICD-10-CM:Z99.12 |

CPT: Current Procedural Terminology.

RxNorm:Medical prescription normalized Medical prescription.

ICD-10-CM: International Classification of Diseases, Tenth Revision, Clinical Modification.
